# Does the Hikikomori Syndrome of Social Withdrawal Exist in Denmark? A Research Request

**DOI:** 10.31662/jmaj.2021-0217

**Published:** 2022-12-23

**Authors:** Jens Peter Eckardt

**Affiliations:** 1 Research Unit, Bedre Psykiatri, Copenhagen, Denmark

**Keywords:** hikikomori, mental health, transcultural psychiatry, social withdrawal

## Abstract

Hikikomori, a severe form of social withdrawal, has long been examined in Japan. Hikikomori-like incidents have also lately been described in many other countries but not in Denmark or any other Scandinavian country thus far. The reason for this is unknown. However, looking at existing research and global attention and its relevance to psychiatric practice today, hikikomori appears as a syndrome that cannot be confined solely to the field of some countries or cultures. Rather, it emerges as a phenomenon that may also concern many aspects of a modern society like the Danish. In the light of considerable quality research on hikikomori in Japan and with increasing international awareness and experiences, the author hereby calls on the health and research community to draw crucial interest to Scandinavian countries, such as Denmark.

Originating in Japan, the hikikomori syndrome―a severe and extreme form of social withdrawal―has become increasingly widespread and achieved global attention among researchers, politicians, and clinicians ^(1), (2)^, given its socioeconomic implications and making it an urgent issue for many governments. However, so far, it has not been described in Denmark. As cases and descriptions of hikikomori in countries, such as Japan, Hong Kong, Oman, France, Italy, Ukraine, Spain, USA, UK, Australia, Bangladesh, Iran, India, South Korea, Taiwan, Thailand, and China, have been reported, it keeps challenging previous narratives of hikikomori as a culture-bound syndrome ^(3)^, in which identification of cases in Denmark and across similar cultures could be observed. So, far, there is a variability in the prevalence of hikikomori in global studies, ranging from 0.87% to 1.2% in Japan (up to 26.66% in Japan’s student population), 6.6% in China, 1.9% in Hong Kong, 2.3% in Korea, 20.9% in Singapore, 9.5% in Nigeria, 2.7% in the United States, and 9% in Taiwan. This variability may, of course, be caused by distinctions in the inclusion criteria, assessment methods, and enrollment strategies across countries and continents.

Hikikomori is a public mental health concern worldwide and the term is usually proposed as pathological social withdrawal or social isolation in the person’s home from all social engagement for at least 6 months, combined with significant functional impairment or distress associated with this isolation ^(4)^. According to Kato et al., hikikomori even tends to co-occur with other psychiatric conditions, and as a comorbidity with other psychiatric disorders, such as schizophrenia and psychotic disorders; depression; social anxiety and other anxiety-related disorders; personality disorders; post-traumatic stress disorder and trauma-related disorders; autism spectrum disorder; other mental illnesses and neurodevelopmental disorders; adjustment disorder; and suicidal risk factors ^(5)^. Even internet gaming disorders and heavy internet and media device use, such as smartphone addiction, have been linked as a crucial contributing factor to the occurrence of hikikomori. However, as stated by researchers, whether psychiatric disorders give rise to hikikomori as a symptom or whether hikikomori is the cause of coexistent psychiatric disorders has not been evidently resolved yet. Despite its complexity and magnitude still being debated, the lack of consensus on diagnostic criteria and a standardized definition for hikikomori, combined with the unclear indication of its global prevalence, its cumulative interest across global cultures and countries is still driven by the emerging field of substantial and widely accepted research.

With proposed operational criteria, assessment tools, and intervention programs, hikikomori should undergo investigation in Denmark as well. Nonetheless, to date, no cross-national studies have yet been conducted in Denmark or any other Scandinavian country. Research designed to identify hikikomori in this European region has overall been completely absent.

The increasing evidence of hikikomori outside Japan gives rise to an urgent request of research in Denmark, especially with overlaps of cultural factors. Such factors include social values and behaviors; increasing levels of stress symptoms among the Danish population; rising numbers of Danish people with psychiatric conditions; social and mental consequences of the coronavirus disease 2019 (COVID-19) pandemic; recent research observations such as loneliness, depressive symptomatology, and suicide ideation in Danish adolescents; associations of loneliness and social isolation with physical and mental health problems among young adults; and rising social disconnectedness among adolescents in Danish high schools.

Considering the factors that may contribute to hikikomori in Denmark, one may look to certain characteristics, pathologies, reactions, and coping strategies within the Danish society. In my opinion, such characteristics of the Danish society or Danish people that may be associated with hikikomori could be factors considering the transformation from a former nuclear family-based society to a society often characterized by a hyper individualized and heavily independent culture or state of mind today. A culture that seems “accelerating, fluid, and unstable” without as many boundaries and obligations as earlier, but at the same time, is strongly digitally structured and designed. A so-called digitally driven civilization and generations mixed with an ever-changing public and interpersonal role of the individual. Even though the Danish society is surrounded by an advanced economic and demographic-sensitive social welfare system, it still might become more perplexing, determining difficulties in recognizing oneself in, for example, the family, interpersonal relationships, civil life, the education system, and work life. One could therefore turn toward social withdrawal as an attempt to get away from these obstacles and complexities, while at the same time affecting the development of specific psychopathologies, such as hikikomori.

It is not unlikely that cases in Denmark would also impact not only the individual’s mental health but also population-level education and workforce stability, including the school context and family relationships. This may be an important psychopathology with overlaps with previously reported results of hikikomori related to family factors, the COVID-19 situation, loneliness, and a wide coexistence with common mental disorders, such as autism spectrum disorders, social anxiety, depression (also modern type depression), internet gaming disorders, problematic internet use, and schizophrenia.

Hikikomori is still a hidden epidemic as a modern society syndrome in many countries, and to grasp its Danish relevance and accurate awareness of the current situation in Denmark, the Scandinavian countries should be included in future studies. It seems important to explore the existing multidimensional assessment systems for hikikomori and to apply these useful insights in a future evidence-based evaluation, early detection program, and treatment, to obtain a better understanding of this phenomenon in a Danish context compared to other countries, such as Japan.

In the light of substantial quality research on hikikomori in Japan, and with increasing international interest and experiences, there is now a call to the health and research community to also draw urgent attention to Scandinavian countries, such as Denmark. Hikikomori has now crossed the limits of a culture-bound phenomenon to become an increasingly relevant concern in Denmark too. Thus, there is a clear need for cross-national and proactive research and efforts to respond to the widespread consequences of hikikomori, which is probably the first sign of a greater disruption in modern society in general.

It is hoped that future cross-national research in a Danish context also will add to a more comprehensive understanding of the existing relevant knowledge and help reveal new aspects of this critical condition. For these abovementioned reasons, continuous global cooperation and research on the hikikomori syndrome will be of vital importance.

## Article Information

### Conflicts of Interest

None

### Author Contributions

Mr. Jens Peter Eckardt wrote and prepared the manuscript for submission.
